# Barriers to Sunscreen Use in a Diverse Patient Population: A Survey-Based Study

**DOI:** 10.7759/cureus.107756

**Published:** 2026-04-26

**Authors:** Hannah Leibowitz, Kevin Wang, Janet Choi, Isabelle Ilan, Shaun Wu, Raquel Klinger, Hailey Konisky, Benedict Wu, Kseniya Kobets

**Affiliations:** 1 Division of Dermatology, Albert Einstein College of Medicine, Bronx, USA

**Keywords:** barriers, medically underserved population, patient perspectives, photoprotection, skin of color, spf, sunscreen

## Abstract

Patients with skin of color (SOC) are more likely to be diagnosed with skin cancer at an advanced stage and with a worse prognosis compared with non-Hispanic White (NHW) patients. This disparity is often attributed to lower reported rates of sun-protective behaviors among adults from racial and ethnic minority groups. This cross-sectional questionnaire study examined barriers to daily sunscreen use and key factors influencing sunscreen purchasing decisions among a predominantly SOC patient population. Forgetting to apply sunscreen was the most frequently reported barrier among sunscreen users, followed by the perception that sunscreen was unnecessary because of spending most of the day indoors. Female patients were more likely than male patients to report no barriers or daily use and were also more likely to use tinted sunscreen. Younger patients placed greater importance on price and product feel or appearance when selecting sunscreen, whereas non-Hispanic Black patients were less likely to consider how the product feels or looks on the skin. Hispanic/Latino patients were less likely than NHW patients to prioritize broad-spectrum protection, and male patients were less likely than female patients to consider specific sun-protection factor levels. These findings highlight distinct behavioral, structural, and cultural factors influencing sunscreen use among SOC patients. Dermatologists play a critical role in addressing misconceptions, counseling patients on affordable and cosmetically suitable options, and promoting inclusive education about sun protection. Understanding these differences can help clinicians deliver culturally responsive recommendations and support targeted interventions to improve sunscreen adherence.

## Introduction

Individuals with skin of color (SOC) face disproportionately higher rates of advanced-stage skin cancer diagnoses and poorer prognoses than their non-Hispanic White (NHW) counterparts [1]. Since sun-protective behaviors (SPBs), such as sunscreen use, reduce the incidence of melanoma and nonmelanoma skin cancers, this disparity is often attributed to lower reported rates of SPBs among adults from racial and ethnic minority groups compared with NHW adults [1,2].

Individuals who do not use sunscreen often encounter multiple barriers, including limited access to clear, accurate educational resources, which may lead to misconceptions that melanin offers complete protection against ultraviolet (UV) damage in SOC [3]. Structural obstacles such as time constraints, cost, and limited product availability can further hinder sunscreen use [3,4]. As individuals with SOC comprise a smaller proportion of the general population and are underrepresented in many dermatologic study cohorts, findings from prior studies may not be fully generalizable to SOC populations. In addition, much of the existing literature is more than a decade old and may not reflect recent shifts in sunscreen-related barriers.

To address this gap, our study at dermatology clinics within Montefiore Medical Center, Bronx, USA, examined barriers to sunscreen use among a predominantly SOC patient population. The objectives of the study are to clearly identify barriers to daily sunscreen use and the factors that contribute to sunscreen purchasing habits in our patient population. Since dermatologist recommendations and counseling strongly influence patients’ sunscreen habits, our goal is to inform physicians about the specific barriers faced by racially and ethnically diverse populations [5,6]. We also aim to support patient advocacy by underscoring the need for manufacturers and public health organizations to develop interventions that address the unique barriers to effective SPBs in SOC populations.

## Materials and methods

This study was conducted at dermatology clinics affiliated with Montefiore Medical Center in the Bronx, USA, over a one-month period in October 2024. Patients aged 13 and older who were English-speaking were eligible. Patients who had difficulty understanding the survey or were unable to provide informed consent were excluded.

Participants were recruited using consecutive sampling. Written informed consent was obtained from all participants prior to survey completion. The study was approved by the Institutional Review Board of Montefiore Medical Center (IRB #2024-16200).

Data were collected via a piloted paper-based survey developed originally by the authors and reviewed by experts to assess: 1) barriers to sunscreen use and 2) factors considered most important when purchasing sunscreen. For both questions, participants were instructed to select all options that applied. The full survey is included in the Appendix.

Demographic variables collected included age, sex, race/ethnicity, skin type, personal and family history of skin cancers, and chief complaint. These factors were considered potential confounders in the analyses.

Descriptive statistics were used to summarize survey responses. Multivariable logistic regression models were employed to assess associations between demographic characteristics and each individual response. Multiple testing correction was performed using the Benjamini-Hochberg procedure at a false discovery rate of 0.05. All statistical analyses were conducted using R software version 4.4.1 (R Foundation for Statistical Computing, Vienna, Austria).

## Results

As shown in Table 1, of 160 sunscreen users, 155 provided responses about barriers to use. Among sunscreen users, forgetting to apply sunscreen was the most reported barrier, followed by those who felt sunscreen was unnecessary due to spending most of their time indoors.

**Table 1:** Barriers to sunscreen use The total number of respondents is 155 ^*^Respondents can choose more than one response

Response	n (%)^*^
It feels too thick	20 (12.9%)
My skin is too oily	16 (10.3%)
My skin is too sensitive	17 (11.0%)
I often forget to apply	49 (31.6%)
I do not need it since I am indoors most of the time	34 (21.9%)
No barriers, I wear sunscreen daily	55 (35.5%)

As shown in Table 2, 149 patients provided responses about the factors that most influenced their sunscreen purchases. The sun-protection factor (SPF) number was the most selected consideration (107; 71.8%), followed by how the product feels or looks on the skin (78; 52.3%). Other key factors included price (52; 34.9%) and broad-spectrum protection (39; 26.2%). To further explore differences by race and ethnicity, we examined reported barriers to sunscreen use and key purchasing factors within each demographic group.

**Table 2:** Key factors when purchasing sunscreen The total number of respondents is 149 ^*^Respondents can choose more than one response SPF: sun-protection factor

Response	n (%)^*^
Price	52 (34.9%)
Water resistance	46 (30.9%)
Broad spectrum	39 (26.2%)
SPF number	107 (71.8%)
Tint/color of sunscreen	20 (13.4%)
Ingredients	43 (28.9%)
How it feels/looks on the skin	78 (52.3%)

Logistic regression analyses revealed several significant findings, with no multicollinearity observed among predictors. Analysis revealed that Hispanic/Latino patients were significantly less likely than NHW patients to report forgetting to apply sunscreen (OR = 0.20; 95% CI = 0.08-0.50). Additionally, male patients were less likely than female patients to report having no barriers to sunscreen use (OR = 0.20; 95% CI = 0.06-0.49).

Among factors influencing sunscreen purchases, older patients were less likely to consider price (OR = 0.96; 95% CI = 0.94-0.98) and how the product feels or looks on the skin (OR = 0.95; 95% CI = 0.93-0.97) important. Hispanic/Latino patients were less likely than NHW patients to prioritize broad-spectrum protection (OR = 0.19; 95% CI = 0.07-0.53). Finally, non-Hispanic Black (NHB) patients were also less likely than other racial groups to prioritize how the sunscreen feels or looks on the skin (OR = 0.13; 95% CI = 0.04-0.45). Post hoc analysis reveals that male patients were significantly less likely than female patients to try tinted sunscreen (OR = 0.13; 95% CI: 0.02-0.48), with a trend suggesting that NHB participants were also less likely than NHW participants to do so (OR = 0.27; 95% CI: 0.06-1.08; p = 0.072).

## Discussion

This study provides insight into sunscreen use behaviors and perceived barriers among SOC patients, highlighting how these barriers vary by demographic characteristics. By directly asking patients about specific obstacles such as sunscreen texture, skin oiliness, forgetfulness, and time spent indoors, we identified patterns that reflect both individual preferences and broader structural and cultural influences on SPBs.

Hispanic/Latino individuals were significantly less likely than NHW patients to report forgetting to apply sunscreen. These differences suggest that lower reported sunscreen use in this population may reflect broader structural challenges and culturally informed decisions rather than a lack of awareness. For example, individuals with SOC may receive conflicting public health messages about whether sun exposure is harmful or necessary for maintaining vitamin D levels, given melanin's protective role [7,8]. This finding underscores the need for dermatologists to address systemic and informational gaps during patient encounters to encourage SPBs that may contribute to long-term positive health outcomes.

Female patients were significantly more likely than male patients to report no barriers to sunscreen use or wearing sunscreen daily. However, existing literature indicates that male patients are less likely to use sunscreen overall [9]. In this study, men were also less likely to use tinted sunscreen, with a trend toward lower use among NHB participants compared with NHW participants. NHB patients were also less likely to consider texture or appearance as a purchasing factor, which may reflect the limited availability of cosmetically suitable products for darker skin tones and illustrates how intersecting gender and racial factors create barriers for men of color. Additional barriers for men include lower engagement with skincare routines and social norms [10,11]. Future research and tailored patient education should consider these intersecting barriers when promoting SPBs among SOC men.

SPF number was the most frequently cited purchasing consideration, followed by how the sunscreen feels or looks on the skin. Younger patients were more likely to consider price and product feel or appearance, suggesting that affordability and cosmetic preferences may drive adherence in this group. Hispanic/Latino patients were less likely than NHW patients to prioritize broad-spectrum protection, which is critical in preventing UV-induced skin damage [1]. This may reflect lower awareness of the importance of broad-spectrum protection, highlighting opportunities for targeted education during dermatology visits.

Previous studies have shown that online sunscreen education materials are often written above the American Medical Association’s recommended reading level and rarely include tailored guidance for SOC audiences [3]. Findings from this study may inform improved outreach and education strategies in both clinical settings and community-based initiatives [2].

This study has several limitations that should be addressed, but do not undermine the validity or contribution of the work. Conducted at a single center in the Bronx, the findings may not be generalizable to other regions. Limiting the sample to English speakers may have excluded key perspectives, and consecutive sampling may not have captured patients who are less engaged in healthcare. Additionally, while the survey captured several common barriers and purchasing factors, it may not have encompassed the full range of residual confounding factors, such as psychosocial, structural, and cultural influences on sunscreen use.

## Conclusions

This study highlights how sunscreen use behaviors and perceived barriers differ across a diverse patient population, particularly among individuals with SOC. The findings suggest that sunscreen use is influenced by a complex interplay of personal habits, cultural beliefs, product accessibility, and the cosmetic limitations of available sunscreen formulations. Differences across racial, ethnic, age, and gender groups emphasize that low sunscreen use among SOC patients is rarely due to a single factor and often reflects broader informational and structural challenges. These results reinforce the importance of clinician-led education that addresses misconceptions and acknowledges the need for affordable and cosmetically suitable products for all skin tones.

By recognizing these varied influences, dermatologists can better tailor counseling to meet the needs of diverse patient populations and help reduce disparities in UV-related skin disease. Continued research should explore barriers among non-English speaking groups and evaluate educational interventions that promote inclusive and practical sun-protection strategies.

## Figures and Tables

**Figure 1:**
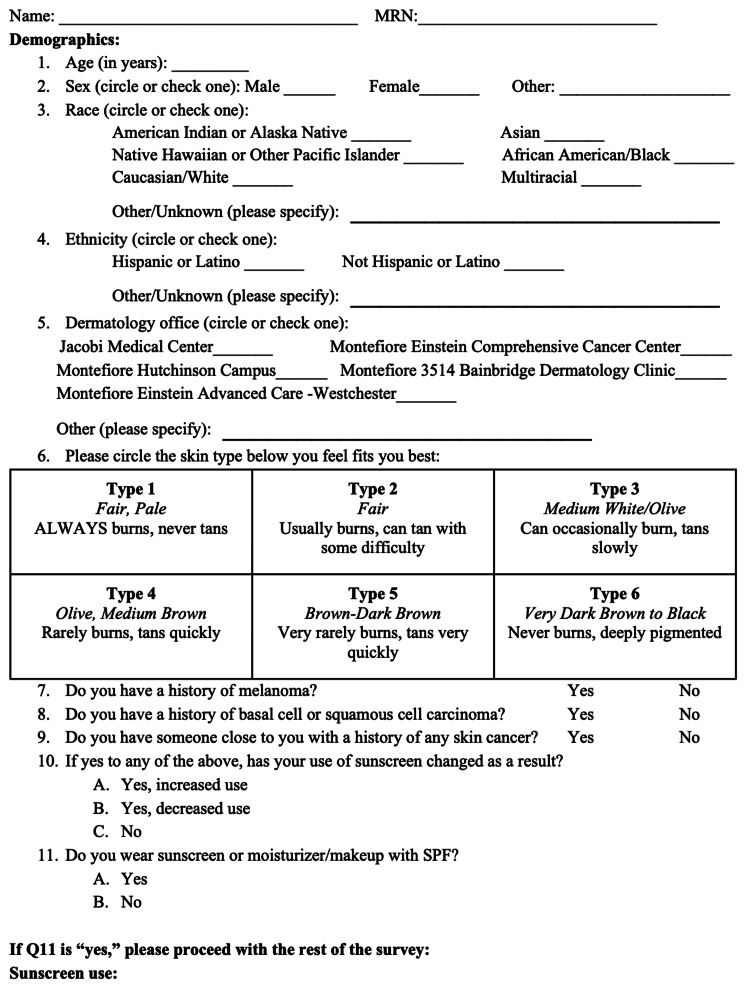
Survey page 1/4

**Figure 2:**
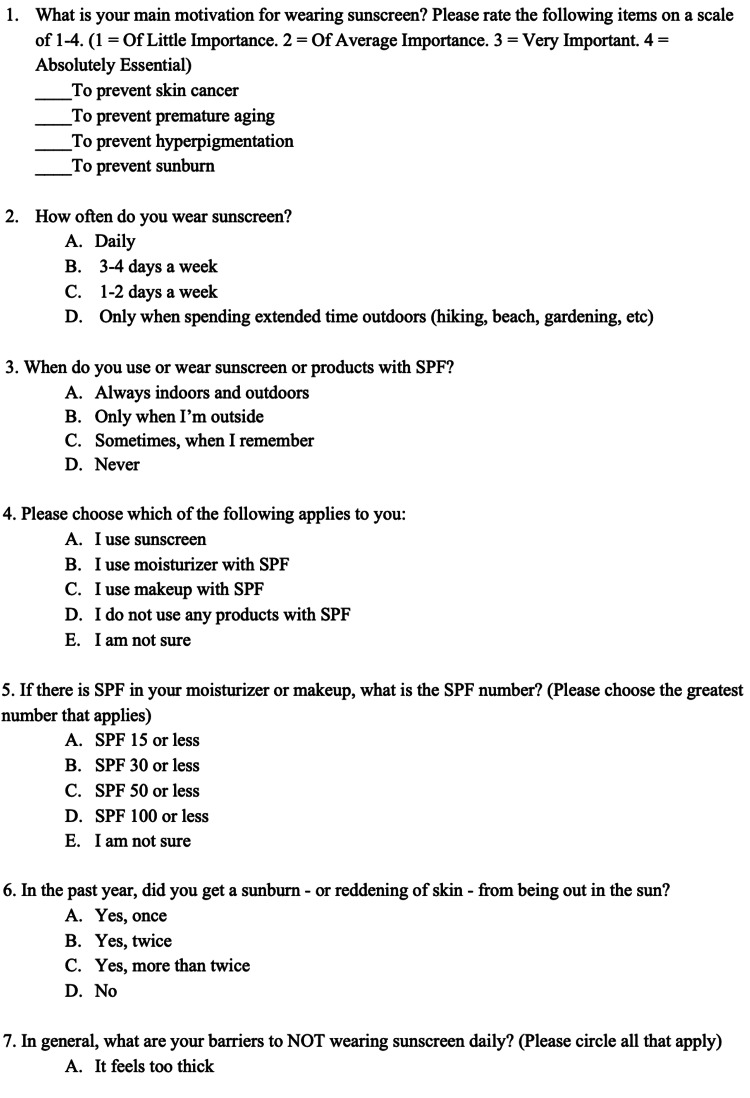
Survey page 2/4

**Figure 3:**
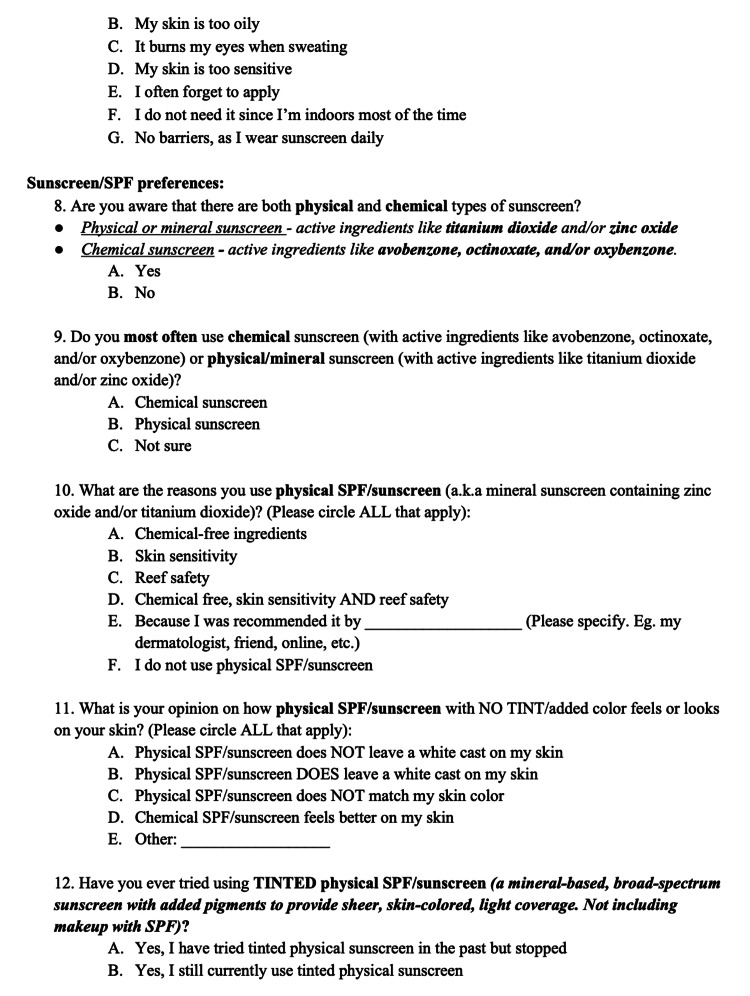
Survey page 3/4

**Figure 4:**
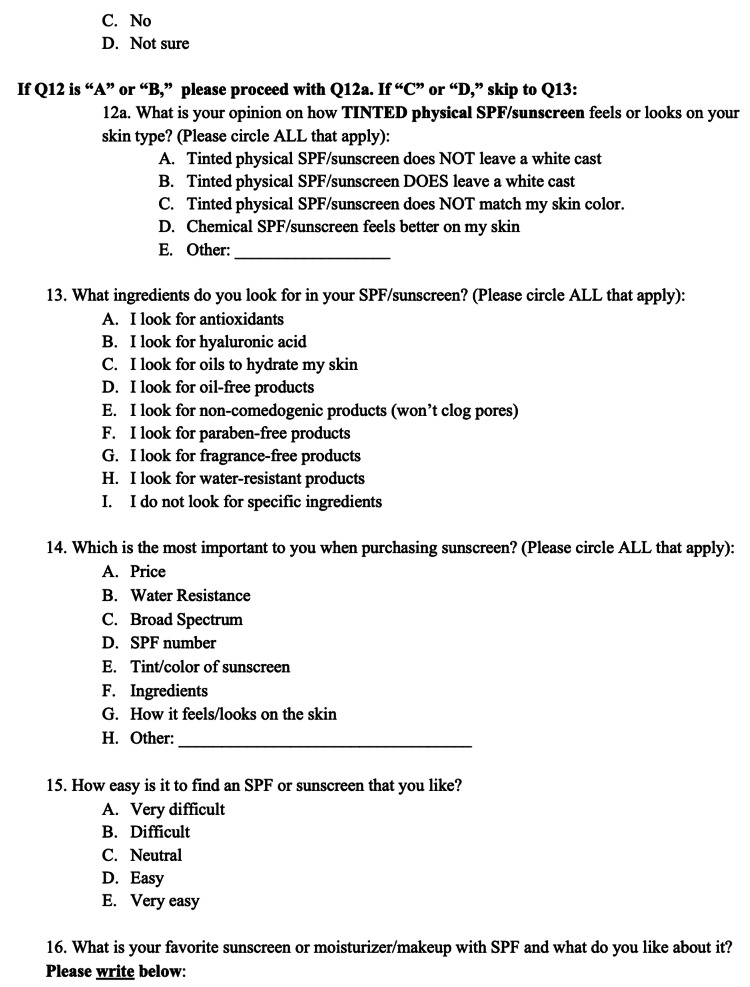
Survey page 4/4
